# The impact of telemental health interventions on maternal mental health outcomes: a pilot randomized controlled trial during the COVID-19 pandemic

**DOI:** 10.1080/21642850.2022.2155167

**Published:** 2022-12-28

**Authors:** Sarah Naja, Rowaida Elyamani, Mohamad Chehab, Mohamed Ali Siddig Ahmed, Ghidaa Babeker, Ghinwa Lawand, Rajvir Singh, Nada Adli, Tagreed Mohamad, Iheb Bougmiza

**Affiliations:** a Community Medicine Department, Hamad Medical Corporation, Doha, Qatar; b Community Mental Health Services, Hamad Medical Corporation, Doha, Qatar; c Obstetrics and Gynecology Department, Hamad Medical Corporation, Doha, Qatar; d Cardiology Research Department, Hamad Medical Corporation, Doha, Qatar; e Community Medicine Department, Primary Health Care Corporation, Doha, Qatar

**Keywords:** Maternal mental health, EPDS, COVID-19, telemental health intervention

## Abstract

**Background:**

The lethal potential of COVID-19 was often emphasized and repeatedly brought to the attention of pregnant women, leading to a higher level of anxiety, depression, and COVID-19-specific phobia among this population. Furthermore, legislation forced social distancing and isolation to interrupt the infection cycle. Together these factors resulted in higher maternal mental health distress requiring intervention. Nevertheless, there is a lack of evidence regarding the impact of low-intensity psychosocial telemental interventions on maternal mental health outcomes. Therefore, the objective of this pilot study is to assess the efficacy of video low-intensity psychosocial telemental maternal intervention on COVID-19-specific phobia, antenatal depression, and anxiety among pregnant women. We hypothesized that the intervention arm would be superior to the control arm. A parallel design randomized interventional controlled trial with 1:1 randomization was conducted at the Women Wellness and Research Center. We enrolled fifty-eight pregnant women in their second trimester who spoke English or Arabic. We assessed antenatal anxiety, depression, and Covid-19-specific phobia at baseline (T_0_), and thirty-three pregnant women completed the follow-up after four weeks (T_1_). Pregnant women receiving psychotropic medications and follow up in mental health services were excluded.

**Results:**

A low-intensity psychosocial telemental maternal session helps reduce antenatal anxiety. We found statistically significant differences in antenatal anxiety scores between the intervention (2.4 ± 2.2) and control (4.2 ± 1.6) groups (*p* = 0.013) with a large effect size of Hedges’ g value (0.96, 0.22–1.74). The absolute risk reduction was 27.27 percent. However, the intervention had no statistically significant effect on reducing antenatal depression or COVID-19-specific phobia.

**Conclusions:**

Low-intensity psychosocial telemental maternal sessions effectively reduce antenatal anxiety. While our findings are promising, further RCTs are needed to replicate these findings.

**Trial registration:**

2a-ClinicalTrials.gov identifier: NCT04594525.. Registered on 20/October/2020; updated 9/March/ 2022. Available from: Maternal Telemental Health Interventions in Response to Covid-19* – Full Text View – ClinicalTrials.gov.

## Background

COVID-19 had a sweeping effect on pregnant mothers diagnosed with the disease (United Nations Children’s Fund [UNICEF], 2020). In addition to the normal challenges of pregnancy, such as attending regular antenatal check-ups and accessing health services**,** evidence showed that pregnant women with confirmed COVID-19 cases had higher intensive care admission rates, invasive ventilation, and increased risk of maternal death compared to pregnant women without Covid-19 infection (United Nations Children’s Fund [UNICEF], 2020; Allotey et al., 2020). In addition to the potential physical health complications of the infection, the pandemic introduced multifaceted stresses, such as isolation, poor social support, and fear of getting infected; these factors were silently triggering maternal mental health diseases (Chen et al., 2020; Farewell et al., 2020; Rashidi Fakari & Simbar, 2020; Saccone et al., 2020).

Early during the COVID-19 pandemic, several studies in China, Pakistan, Iran, and Canada conveyed concern about a possible trend in maternal mental health distress. These studies reported an increase in antenatal depression and anxiety symptoms (Effati-Daryani et al., 2020; Lebel et al., 2020; Shahid et al., 2020; Wang et al., 2020; Wu et al., 2020). Furthermore, cumulative evidence from two meta-analyses found that the rate of antenatal anxiety and depression heightened during the pandemic (Kamran et al., 2020; Tomfohr-Madsen et al., 2021). Similarly, in Qatar, a preliminary study estimated a rise in the percentage of antenatal anxiety and depression symptoms compared to data before the COVID-19 pandemic. Specifically, Qatar’s prevalence of antenatal depression increased by 10% (24% vs. 34.4%), and antenatal anxiety increased by 22.8% (16.4% vs. 39. 2%) (Farrell et al., 2020; Naja et al., 2019; Naja et al., 2020).

The infectious pandemic led to a rise in risk perception that provoked the emergence of fear, which is a rational reaction to life-threatening events. However, the pandemic provoked a unique kind of stress, COVID-19-specific phobia, an abnormal and irrational fear in which an individual is uncontrollably worried about contracting COVID-19, even without any specific physical symptom. Fear of disease or ‘Nosophobia’ is a psychological disorder that could disrupt a person's daily life (Heiat et al., 2021). The fear of COVID-19 augmented antenatal anxiety and depression among pregnant women, consequently disrupting maternal mental health well-being (Fitzpatrick et al., 2020; Giesbrecht et al., 2022; Underwood et al., 2016). Furthermore, it increased the odds of preterm delivery, C-section, and neurodevelopmental disorders in newborns (Dunkel Schetter & Tanner, 2012; Roseboom et al., 2021; Waters et al., 2014). Thus, maternal mental health is a public health priority given its detrimental outcomes.

Globally, international organizations were aware of the impact of untreated maternal mental illness on expecting mothers and children and the cost–benefit and cost-effectiveness of the intervention, which led the United States Task Force (USPSTF) to support screening for low mood during pregnancy (Bauer et al., 2022; United State Preventive Services Task Force [USPSTF], 2019). Specifically, it was evident that face-to-face Cognitive Behavioural Therapy (CBT) revealed a protective effect (OR 0.64; 95% CI [0.53–0.75]) and a preventive effect (OR 0.39; 95% CI [0.17–0.60], p 0.001) (Sockol, 2015). Consequently, guidelines recommended cognitive behavioural therapy as a first-line option to treat low mood in pregnant women as it also mitigates the incidence of postpartum depression (Molenaar et al., 2018; Clatworthy, 2012).

Qatar’s maternal mental health services are still in an early development phase. Most antenatal care systems worldwide do not provide any structured maternal mental health screening, prevention, or even referral for treatment. However, Qatar National Strategy supports promoting maternal mental health in the population (Ministry of Public Health [MOPH], 2022).

During the COVID-19 pandemic, efforts were made to ensure the continuity of mental health care while respecting the social distancing enforced by the pandemic through virtual consultations and helplines (Wadoo et al., 2020). Telecommunications can ease fear, anxiety, and depression in the general population, as reported by a systematic review of randomized controlled trials (RCTs) (Abraham et al., 2021). However, no randomized control trials assessing the efficacy of maternal low-intensity psychosocial interventions delivered via telemental health channels on antenatal anxiety, depression, and COVID-19-specific phobia among pregnant women in Qatar have been performed.

## Objectives

The objective is to measure the efficacy of low-intensity psychosocial video-based consultations on antenatal mental health outcomes by hypothesizing that the intervention arm will be superior to the control arm. In addition, this research tests the null hypothesis stating that pregnant women who received telemental low-intensity psychosocial video consultations have the same antenatal psychological distress (antenatal depression, antenatal anxiety, and COVID-19-specific phobia) compared to pregnant women who did not receive the same intervention during the COVID-19 pandemic.

The following research question will be addressed: What is the efficacy of telemental low-intensity psychosocial video consultations on antenatal mental health outcomes?

## Methods

### Study population and recruitment

#### Study design and setting

This is a randomized, controlled, parallel trial of the impact of video-based low-intensity psychosocial interventions on antenatal psychological wellbeing among pregnant women attending the antenatal clinics at the Women Wellness and Research Center (WWRC) in Qatar.

The trial was approved by the Hamad Medical Research Centre, registration MRC 01-20-1129, and registered in clinicaltrials.gov: 2a-ClinicalTrials.gov NCT04594525. The study was registered on 20/October/2020 and updated on 9/March/2022. Trial information is available from: Maternal Telemental Health Interventions in Response to Covid-19*– Full Text View – ClinicalTrials.gov.

The trial included two phases: training of data collectors and screening for eligibility before recruitment. First, the principal investigator trained the psychologist on the WHO materials. Specifically, the training included formal classroom training and hands-on practice, including the practical aspects of using VSee technology.

The WWRC is a governmental hospital that provides secondary health care services to women of reproductive age in Qatar; it provides a continuum of antenatal and postnatal reproductive care. The service users’ cohort represents the community in Qatar as it accommodates patients of various social and economic classes. The average number of patients attending early pregnancy clinics can reach 20 per shift. The WWRC’s antenatal clinic attendance rate was as high as 70% of the total live births during 2019 in Qatar (about 12,896 pregnant women). Maternal mental health support classes were not available at the WWRC during the time of the study; they were provided by a tertiary care hospital (Hamad Medical Corporation [HMC], 2022).

#### Sampling methods or strategy

The eligible participants included pregnant women registered at the WWRC above 18 years old. The inclusion criteria were pregnant women in their second trimester who accepted video consultation and verbally consented to participate in the study. The exclusion criteria included pregnant women diagnosed with psychiatric illness and who had follow-up appointments with public mental health services and participants receiving mood stabilizers or antidepressants. Furthermore, participants who did not speak and understand English and Arabic were excluded.

The obstetric team extracted a list of potential participants from the hospital’s electronic records. Initially, the records were screened, then participants were selected randomly by a random generator from the eligible list to complete all the screening questions via a telephone interview. Finally, a minimum of three phone calls were made to potential participants, and if there was no response, then they were removed from the list.

Before commencing the video consultation sessions, random allocation of the participants (1:1) into the intervention and control groups was performed through excel, and the group allocations were concealed. The authors had the participant’s code, allocated arm, and phone number in a sealed envelope labelled with either the letter A or B. The cohort of participants was followed from the screening phase until the end of the interventional sessions (T_1_-session 2).

#### Intervention description

The intervention was extracted from the World Health Organization (WHO) ‘Thinking Health’ manual (World Health Organization [WHO], 2015). We offered one-to-one sessions, two sessions of low-intensity psychosocial intervention for each participant; each session lasted up to 45 min, was scheduled four weeks apart, and was delivered through video consultation and the use of the screen-sharing software tool VSee. The VSee platform allows active interaction between the provider and the participant with assured privacy and confidentiality and optimum security against hacking. Furthermore, it is accessible through smartphone applications.

The provider wore professional attire and sat in front of a simple, non-distracting background. The psychologist provided the sessions in one of the community medicine department’s private rooms on the 5th floor, Barwa tower 2, primary health care corporation, where the VSee application is accessed from the computer.

The patients were instructed to schedule their visit when they could be alone in a quiet room and were encouraged to avoid multitasking.

The WHO ‘Thinking Health’ manual consists of evidence-based strategies, including developing an empathetic relationship, stimulating thoughts, behaviour initiation, problem-solving, and involving the family. These elements were applied in two sessions and delivered in English or Arabic language based on the patient’s primary language.

**First session:** The first session, at T_0,_ included an assessment of baseline sociodemographic information, pregnancy-related characteristics, medical history, behavioural factors (physical activity, smoking status and alcohol consumption), possible psychosocial stresses (stressful life events, gender-related stress, financial distress, partner emotional support and COVID-19 exposure) and a psychosocial assessment (antenatal depression, anxiety, and COVID-19 phobia) for the two arms.

In T_0_, the intervention group was introduced to the basic principles of cognitive–behavioural therapy and the importance of active participation, informed about the three areas of healthy thinking (Mother’s personal well-being, Mother-infant relationship, Relationship with people around the mother and infant) and empowered to focus on solutions to problems, identify unhealthy thinking and thoughts, and practice replacing them. In addition, they were encouraged to use a daily health calendar and a mood chart.

**Second session:** The follow-up session at T_1_ was conducted four weeks after the initial visit. The intervention group was offered a second session of low-intensity psychosocial intervention, while the control arm was only offered regular services.

At T_1_, the intervention group was asked to review key messages from the previous session, check the participant’s mood chart and health calendar, and conduct a healthy thinking session. The healthy thinking session focused on preparation for the baby, discussing the mother’s thoughts about her health and identifying unhealthy ideas and statements, including exploring her relationship with the baby and her social circle. Following exploration, reasoning with actions and consequences and then techniques for replacing unhealthy feelings with healthy ones through job aids such as health calendar and mood chart were discussed. The sessions were concluded by ensuring that participants were able to practice healthier patterns of thinking. Post-session, the psychosocial outcomes were assessed (Supplementary-I).

#### Control group description

Pregnant women in the control group were screened for outcomes via the same platform in addition to receiving the standard care. The standard care at the Women Wellness and Research Center includes the standard perinatal care. However, it does not include any mental health interventions or treatment.

#### Sample size and participants

We utilized a 95% level of confidence interval (CI), 5% error rate with 80% power to test the null hypothesis. The sample size calculation followed the OpenEpi® software version 3.01 formula (Open Epi, 2021):

n1=(Zα/2+Zβ)2∗(p1(1−p1)+p2(1−p2))/r(p1−p2)2andn2=r∗n1

where *n*1 = number of exposed (intervention group) and *n*2 = number of unexposed (control group); *Zα*/2 = normal standard deviation for the two-tailed test based on the alpha value (relays to the confidence interval) [alpha = 0.05]; *Zβ* = normal standard deviation for the one-tailed test based on the beta-value (relays to the power level) [beta = 0.2]; *r* = ratio of unexposed to exposed = 1; *p*1 = proportion of exposed with the outcome: incidence of perinatal depression in intervention arm: 34% (an effect of 34% and 69% after delivery was reported by two papers) (United State Preventive Services Task Force (USPSTF), 2019; THERA PLATEFORM); *p*2 = proportion of unexposed with the outcome: incidence of perinatal depression in the control arm: 70% (due to lack of similar local studies; hence, the estimate p1 was set to 70%). The sample size desired for each arm is 29 for a total of 58 participants.

### Research protocol

TM screened the records, and GL obtained verbal informed consent following a telephone script in the screening phase. GL then asked three screening questions (adverse history of mental health, use of mental health service, acceptance to open video for consultation). RE generated the random allocation sequence, SN enrolled the participants and assigned the participants to the interventions. The principal investigator delivered the matching envelopes that contained the consent forms and data collection sheets to the psychologist in the recruitment phase. Next, the psychologist opened the envelope to find the patient code, the allocated group and the phone number to book the date and time for the video interview. Finally, each participant received a message on their cell phone containing the link to the Vsee platform to access the session at the appropriate time.

The psychologist secured consent before each video conference. Data were obtained at different time points (T_0_ and T_1_) using paper-based screening items, structured interviews, and mental health self-reporting tools. All patient-related information was coded to secure patient protection. In addition, the team ensured proper documentation on a screening/enrolment and randomization log.

Blinding was done at two levels: at a group level for a data analyst to reduce performance and ascertainment bias and a data entry level by providing a unique code for each subject before entering the analysis data. The mitigation plan indicated a low-risk total score was 24, and the risk assessment showed that the benefits outweigh the risk.

### Data collection tools

After giving consent, participants were asked about their sociodemographic characteristics, whether the pregnancy was intended, pregnancy-related factors, history of unfavourable pregnancy outcomes, Covid-19 status, smoking, alcohol use, fitness score, life stressors, and medical history, including mental health history. The independent variables included all the biopsychosocial factors and Covid-19 exposure history assessed through a structured interview-based tool.

The investigator reviewed all participants’ pregnancy-related history, including medical history and medications. Following the interviewer-administrated questionnaire, psychological assessments were assessed using the Edinburgh Postnatal Depression (EPDS) tool, and Covid-19-specific phobia; then scores were computed for each participant. Participants who expressed thoughts of self-harm (item 10) were referred to the emergency department of the HMC hospitals for an urgent assessment by a specialist in mental health services following the corporate policies. The structured interview was tested for its face, translation, and content validity by experts (content validity 90%). All tools are in English and Arabic language.

*Edinburgh Postnatal Depression (EPDS-10*) is a validated tool of bi-constructs. It was tested in Qatar and was demonstrated to be a reliable screening tool for antenatal depression with a cut-off of score of 13 that showed high sensitivity and specificity (Naja et al., 2019). Furthermore, it demonstrated a pooled sensitivity of 0.80 and a pooled specificity of 0.81 (Chorwe-Sungani & Chipps, 2017). Moreover, the anxiety subscale of *EPDS-3A* was identified to have acceptable sensitivity and specificity based on a systematic review of antenatal anxiety tools with a cut-off of five (Naja et al., 2020).

A *COVID-19-specific phobia scale* of seven items was used to assess the level of fear of the emerging virus. The possible responses ranged on a five-item Likert-type scale from strongly disagree to agree strongly. The total scores ranged from 7 to 35; the higher the score, the greater the phobia of COVID-19. Validation of the tool revealed good internal consistency (*α* = 0.82); however, there was no approved cut-off point (Ahorsu et al., 2020). As the mean, median, and mode are equal, we used the median as a cut-off point of seven to dichotomize the data.

### Analysis

The statistical analyses were performed using IBM SPSS version 25. Cross-tabulations were used to examine the participants’ demographic information, pregnancy-related characteristics, and stress-related factors, including COVID-19 exposure status stratified by group. We used an independent sample t-test and paired t-test to compare the scores before treatment with the those reported after treatment. A chi-square test and Fishers’ exact test were used to compare nonparametric measurements at inclusion. An intention-to-treat analysis was performed to examine the effect of drop out. The effect sizes were calculated using Hedges’ g. Positive effect sizes reduce symptoms from pre- to post-treatment, with effect sizes of 0.20 small, 0.50 moderate, and 0.80 large (Lakens, 2013). To minimize type-1 errors, we did not correct for the missing values and therefore chose to interpret the results more carefully. Later, antenatal anxiety, depression, and COVID-19 phobia were dichotomized to enable computing the Number Needed to Treat (NNT) and absolute risk difference. Differences in scores were assumed to be statistically significant at a *p*-value of 0.05.

## Results

### Flow chart

Screening for the study was open from May to July 2021, and recruitment was from September to October 2021. The participants who refused to enrol were mainly multigravida (67.4%) and Qatari nationals (83.5%). Fifty-four pregnant women did not consent to participate, mainly due to very busy schedules or there was no response on the phone despite three calls.

**Figure 1.**
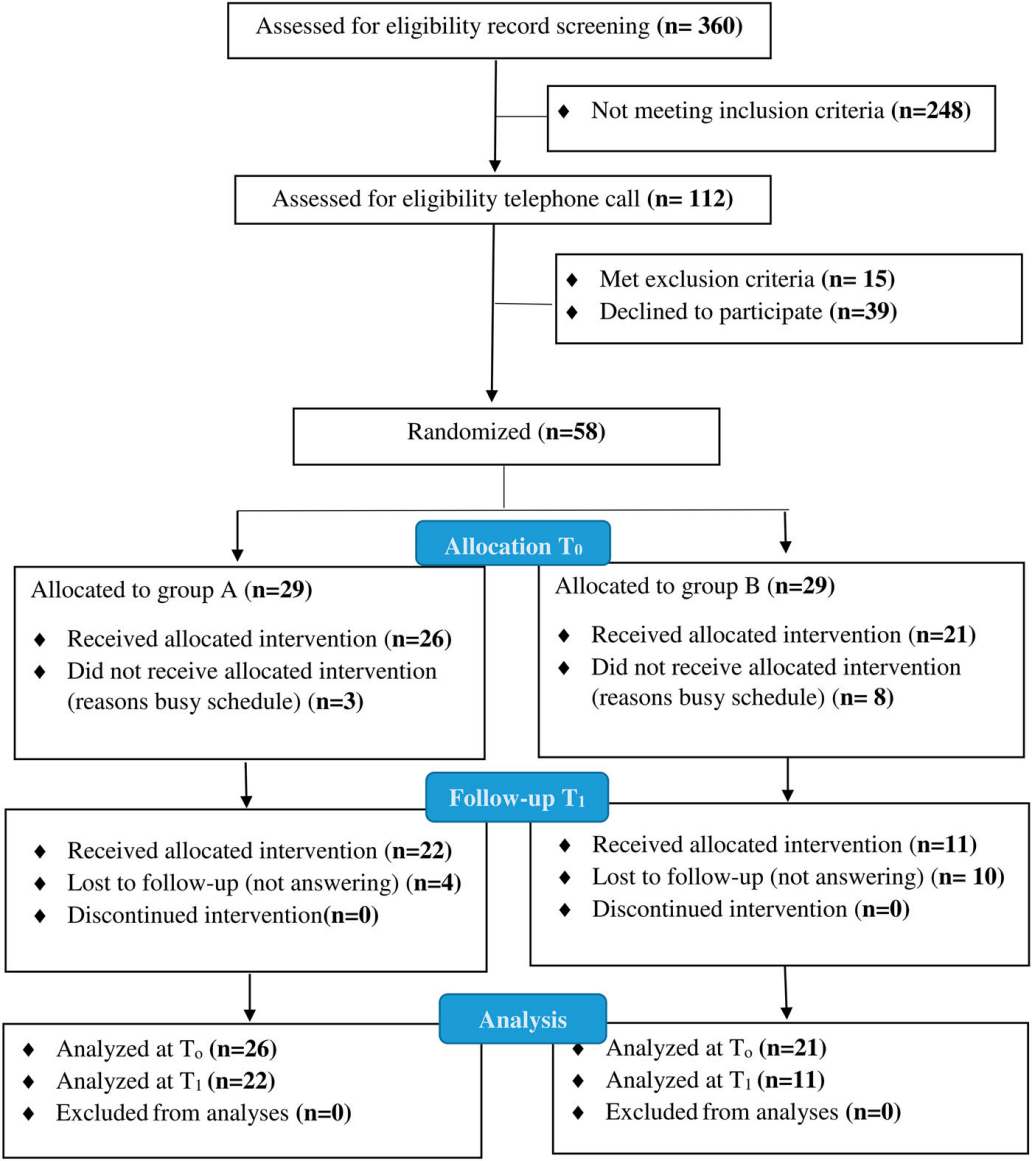
Consort flow chart of the study (*n* = 58).

We followed the Consolidated Standards of Reporting Trials (CONSORT) in displaying the steps of recruitment and randomization. The flow of the research is described in Figure 1.

### Baseline participant’s characteristics

We randomly screened and allocated equal arms, where 29 participants were randomly assigned to each group. At T_0_, 11 participants dropped out. Their mean age was 32, and 60% were Arabs and 54.5% were multigravida. There was no statistical difference in the enrolled pregnant women’s ages between the two groups (*p* = 0.48). The mean age was 32 ± 4.7 years (range, 25–43) for group A and 31 ± 3.8 years (range, 26–39) for group B. Furthermore, the mean gestational age was 20 ± 4 weeks for both groups (*p* = 0.34). Most participants were homemakers with moderate household incomes and higher educational levels. Sociodemographic and pregnancy-related characteristics did not show any statistical differences between the two arms. Few participants had COVID-19 infection, and both groups had similar number of somatic symptoms. On average, group A (Mean 1.3 ± SD 1.4) and group B (Mean 1.2 ± SD 1.1); *p* = 0.87. Furthermore, stress-related factors and fitness levels were not statistically different between the groups as seen in Table 1.

**Table 1.** Baseline characteristics of the two groups of pregnant women attending the Women Wellness and Research Center in Qatar.

	Groups of pregnant women-at T_0_					
	Group A Control		Group B Intervention			Between group Analysis
	*n* = 26	(%)	*n* = 21	(%)	*P*-value	CI
Sociodemographic characteristics						
Nationalities						
Arab	17	(60.7)	11	(39.3)	0.36	1.7 [0.5–5.5]
Non-Arab	9	(47.4)	10	(52.6)		
Educational level						
Secondary education	1	(25.0)	3	(75.0)	0.20	0.21 [0.02–2.4]
Higher education	25	(58.1)	18	(41.9)		
Occupational level						
Housewife	13	(50.0)	13	(50.0)	0.41	0.61 [0.19–1.9]
Employed	13	(61.9)	8	(38.1)		
Household Income						
Up to 10,000 QR	9	(50.0)	9	(50.0)		
10,001–20,000 QR	9	(50.0)	9	(50.0)	0.41	…
> 20,001 QR	8	(72.7)	3	(27.3)		
Pregnancy-related characteristics						
Intended pregnancy						
Yes	9	(42.9)	12	(57.1)	0.12	2.5 [0.77–8.11]
No	17	(65.4)	9	(34.6)		
Nulliparous						
Yes	4	(33.3)	8	(66.7)	0.076	0.29 [0.74–1.17]
No	22	(67.9)	13	(37.1)		
History of abortion						
Yes	8	(50.0)	8	(50.0)	0.59	1.38 [0.41–4.65]
No	18	(58.1)	13	(41.9)		
History of stillbirth						
Yes	2	(66.7)	1	(33.3)	0.68	0.60 [0.05–7.11]
No	24	(54.5)	20	(45.5)		
Gestational Diabetes						
Yes	5	(41.7)	7	(58.3)	0.27	2.11 [0.55–7.95]
No	21	(60.0)	14	(40.0)		
Spontaneous pregnancy						
Yes	24	(55.8)	19	(44.2)		
No	2	(50.0)	2	(50.5)	0.82	1.26 [0.16–9.81]
Possible stressful factors						
Confirmed COVID-19						
Yes	1	(25.0)	3	(75.1)	0.20	4.1 [0.4–43.37]
No	25	(58.1)	18	(49.9)		
Fitness						
Fit	4	(80.0)	1	(20.0)	0.08	…
Active	1	(16.7)	5	(83.3)		
Not fit	21	(58.3)	15	(41.7)		
Acute stressful event						
Yes	2	(33.3)	4	(66.7)		
No	24	(58.5)	17	(41.5)	0.24	2.8 [0.46–17.2]
Gender-related stress						
Not at all	20	(54.1)	17	(45.9)		… .
Very little	3	(60.0)	2	(40.0)		
Sometimes	1	(50.0)	1	(50.0)	0.97	
Great extent	2	(66.7)	1	(33.3)		

*n* = frequency; % = percentage; SD = standard deviation; X = mean; CI = Confidence Interval; Group A = Control; Group B = intervention.

### Between group analysis

There were no significant differences in psychological outcomes between the control and intervention groups at the T_0_ level. However, at T_1_, there was a substantial difference in the antenatal anxiety level between the control and intervention groups. Therefore, the computed Hedges’ *g* value of 0.96 [0.22–1.74] is a large effect size. The intention-to-treat analysis revealed no statistical differences between the control and intervention group in the antenatal anxiety level as seen in Table 2.

**Table 2.** Antenatal depression, anxiety, and COVID-19-specific phobia amongst the two groups of pregnant women attending the Women Wellness and Research Center in Qatar at T_0_ and T_1_.

Sessions	T_0_						T_1_					
Groups	Per-protocol analysis			Per – intention-to-treat analysis			Per-protocol analysis			Per intention-to-treat analysis		
	Group A	Group B		Group A	Group B		Group A	Group B		Group A	Group B	
	Control	Intervention		Control	Intervention		Control	Intervention		Control	Intervention	
	*n* = 26	*n* = 21	Mean difference [CI]; *p*-value	*n* = 29	*n* = 29	Mean Difference [CI]; *p*-value	*n* = 22	*n* = 11	Mean Difference [CI]; *p*-value	*n* = 29	*n* = 29	Mean Difference [CI]; *p*-value
**EPDS scale** X ± SD	11.1 ± 5.1	8.3 ± 6.2	2.8 [−0.4–6.2]; 0.09	11.0 ± 4.8	8.7 ± 5.3	2.3 [−0.4–1.2]; 0.42	9.4 ± 3.5	6.5 ± 5.7	2.8 [−0.3–3.2]; 0.08	9.4 ± 4.1	8.3 ± 5.0	1.1 [−1.1–3.5]; 0.37
**EPDS-3A** X ± SD	3.2 ± 1.7	2.7 ± 2.8	0.46 [−0.8–1.8]; 0.48	4.5 ± 2.2	3.2 ± 1.9	1.3 [−0.2 −0.5]; 0.51	4.2 ± 1.6	2.4 ± 2.2	1.8 [0.41–3.21]; 0.01*	4.2 ± 1.7	3.4 ± 2.0	0.7 [−0.2–1.7]; 0.13
**COVID-19 specific phobia** X ± SD	9.8 ± 4.3	8.0 ± 5.9	1.7 [−3.4 −1.26]; 0.23	9.2 ± 4.4	6.9 ± 5.3	2.3 [1.2–_0.2]; 0.68	8.1 ± 4.7	7 ± 6	1.1 [−2.7 −5.0]; 0.55	7.8 ± 4.7	8.6 ± 4.2	−0.7 [−3.1–1.6]; 0.52

*n* = frequency; % = percentage; SD = standard deviation; X = mean; CI = Confidence Interval; EPDS: Edinburgh Postnatal Depression; EPDS-3A = EPDS anxiety subscale; T_0 _= baseline session; T_1 _= follow-up session; *p* ≤ 0.05*.

### The number needed to treat antenatal depression (NNT)

Table 3 shows that around 33 percent of women in both the control and intervention groups were depressed at T_0_, providing no statistically significant outcome at T_0_. At T_1_, 18.2 percent of the control subjects reported antenatal depression. Similarly, 18.2 percent of experimental subjects had antenatal depression. The absolute risk difference in antenatal depression between the two arms was identical at 0.00 percent per protocol and at 0.2 percent per intention-to-treat analysis. The results show that 50.0 percent of the control subjects and 28.6 percent of the intervention group were anxious at T_0_ with no statistical difference between the groups in this regard. At T_1_, 45.5 percent of the control subjects and 18.18 percent of the experimental subjects had antenatal anxiety. This difference’s 95% confidence interval ranges from −3.59% to 58.13%. The NNT value is four, where about one in every four patients will benefit from the treatment. The absolute risk reduction is 27.27 percent per protocol and at 32 percent per intention-to-treat analysis. The analysis shows that 38.5 percent of the control subjects and 42.9 percent of the intervention group experienced COVID-19-specific phobia with no statistical difference between the two groups at T_0_. At T_1_, 50.0 percent of the control subjects and 27.27 percent of the experimental subjects had COVID-19-specific phobia. The absolute risk reduction is 22.73 percent; the 95% confidence interval ranges from −10.88% to 56.33%. The absolute risk at 32 percent per intention-to-treat analysis.

**Table 3.** Antenatal depression, antenatal anxiety and COVID-19-specific phobia amongst the two groups of pregnant women attending the Women Wellness and Research Center in Qatar at T_0_ and T_1_

	T_0_								T_1_							
	Per-protocol				Per intention-to-treat analysis				Per-protocol				Per intention-to-treat analysis			
Antenatal Depression	Depressed	Not depressed	Between group analysis		Depressed	Not depressed	Between group analysis		Depressed	Not depressed	Between group analysis		Depressed	Not depressed	Between group analysis	
	*n*; (%)	*n*; (%)	Total	*P*-value	*n*; (%)	*n*; (%)	Total	*P* – value	*n*; (%)	*n*; (%)	Total	*p*-value	*n*; (%)	*n*; (%)	Total	*p*-value
Group B-Intervention	7; (33.3)	14; (66.7)	21	0.85	7; (24.1)	22; (75.9)	29	0.76	2; (18.2)	9; (81.8)	11	1	23; (79.3)	6; (20.7)	29	0.73
Group A-Control	8; (33.8)	18; (69.2)	26		8; (27.6)	21; (72.4)	29		4; (18.2)	18; (81.8)	22		24; (82.8)	5; (17.2)	29	
Antenatal Anxiety	Anxious	Not anxious	Between group analysis		Anxious	Not anxious	Between group analysis		Anxious	Not anxious	Between group analysis		Anxious	Not anxious	Between group analysis	
	*n*; (%)	*n*; (%)	Total	*P*-value	*n*; (%)	*n*; (%)	Total	*P*-value	*n*; (%)	*n*; (%)	Total	*p*-value	*n*; (%)	*n*; (%)	Total	*p*-value
Group B-Intervention	6; (28.6)	15; (71.4)	21	0.13	6; (20.7)	23; (79.3)	29	0.09	2; (18.2)	9; (81.8)	11	0.1	6; (20.7)	23; (79.3)	29	0.14
Group A-Control	13; (50.0)	13; (50.0)	26		13; (44.8)	16; (55.2)	29		10; (45.5)	12;(54.5)	22		11; (37.9)	18; (62.1)	29	
Covid-19 Specific phobia	Presence	Absence	Between group analysis		Presence	Absence	Between group analysis		Presence	Absence	Between group analysis		Presence	Absence	Between group analysis	
	*n*; (%)	*n*; (%)	Total	*p*-value	*n*; (%)	*n*; (%)	Total	*p*-value	*n*; (%)	*n*; (%)	Total	*p*-value	*n*; (%)	*n*; (%)	Total	*p*-value
Group B-Intervention	9; (42.9)	12; (57.1)	21	0.20	8; (26.7)	21; (72.4)	29	0.03	3; (27.3)	8; (72.7)	11	0.21	15; (51.7)	14; (48.3)	29	0.42
Group A-Control	10; (38.5)	16; (61.5)	26		16; (55.2)	13; (44.8)	29		11; (50.0)	11; (50.0)	22		18; (62.1)	11; (37.9)	29	

*n*: frequency; %: percentage; T_0 _= baseline session; T_1 _= follow-up session; *p* ≤ 0.05*.

### Within group analysis

There were no significant differences in anxiety and COVID-19-specific phobia within the control groups at the T0 and T1 levels. However, the control group had significant differences in antenatal depression outcomes. The intention-to-treat analysis revealed a substantial difference in the COVID-19-specific phobia within the intervention group level between T0 and T1. Therefore, the computed Hedges’ g value of 0.33 [0.18–0.85] is a small to moderate effect size, as seen in Table 4.

**Table 4.** Within group comparison for RCT.

	Group A Controls						Group B Intervention					
	Per-protocol analysis			Per – intention-to-treat analysis			Per-protocol analysis			Per intention-to-treat analysis		
Groups Sessions	T_0_	T_1_	Within group comparison	T_0_	T_1_	Within group comparison	T_0_	T_1_	Within group comparison	T_0_	T_1_	Within group comparison
	*n* = 26	*n* = 22	Mean difference [CI]; *p*-value	*n* = 29	*n* = 29	Mean difference [CI]; *p*-value	*n* = 22	*n* = 11	Mean difference [CI]; *p*-value	*n* = 29	*n* = 29	Mean difference [CI]; *p*-value
EPDS scale X ± SD	11.5 ± 4.5	9.4 ± 3.5	2.09 [0.7–3.4]; 0.001*	11 ± 4.8	9 ± 4.1	1.5 [0.5–2.6]; 0.05*	7 ± 6.7	6.5 ± 5.7	0.45 [−1.8–2.8]; 0.67	8.7 ± 5.3	8.3 ± 5.0	0.4 [−0.1–1.0]; 0.17
EPDS-3A X ± SD	4.8 ± 2.1	4.2 ± 1.6	0.54 [−0.09–1.1]; 0.09	4.5 ± 2.2	4.2 ± 1.7	0.3 [−0.1–0.8]; 0.2	2.5 ± 2	2.4 ± 2	0.09 [−0.8–1.01]; 0.83	3.2 ± 1.9	3.4 ± 2	−0.2 [−0.6–0.12]; 0.18
COVID-19 specific phobia X ± SD	10.6 ± 4.1	8.1 ± 4.1	2.4 [0.7–4.1]; 0.06	9.2 ± 4.4	7.8 ± 4.2	1.2 [−0.1–2.8]; 0.08	7 ± 6.3	6 ± 6.0	−0.9 [−2.6–0.87]; 0.28	8.6 ± 4.7	6.9 ± 5.3	1.72 [2.7–0.7]; 0.001*

*n* = frequency; % = percentage; SD = standard deviation; X = mean; CI = Confidence Interval; EPDS: Edinburgh Postnatal Depression; EPDS-3A = EPDS anxiety subscale; T_0 _= baseline session; T_1 _= follow-up session; p ≤ 0.05*.

Table 5 shows no significant differences within control groups at T0 and T1. The intention-to-treat analysis revealed a substantial difference in the COVID-19-specific phobia within the intervention group level between T0 and T1.

**Table 5.** Antenatal depression, antenatal anxiety and COVID-19-specific phobia within the two groups of pregnant women attending the Women Wellness and Research Center in Qatar at T_0_ and T_1_.

	Group A Control								Group B Intervention							
	Per-protocol				Per intention-to-treat analysis				Per-protocol				Per intention-to-treat analysis			
Antenatal Depression	Depressed	Not depressed	Within group comparison		Depressed	Not depressed	Within group comparison		Depressed	Not depressed	Within group comparison		Depressed	Not depressed	Within group comparison	
	*n*; (%)	*n*; (%)	Total	*P*-value	*n*; (%)	*n*; (%)	Total	*P* – value	*n*; (%)	*n*; (%)	Total	*p*-value	*n*; (%)	*n*; (%)	Total	*p*-value
T_0_	8; (30.8)	18; (69.2)	26	0.31	5; (17.2)	24; (82.8)	29	0.34	7; (33.3)	14; (66.7)	21	0.44	7; (24.1)	22; (75.9)	29	0.75
T_1_	4; (18.2)	18; (81.8)	22		8; (27.6)	21; (72.4)	29		2; (18.2)	8; (88.8)	11		6; (20.7)	23; (79.3)	29	
Antenatal Anxiety	Anxious	Not anxious	Within group comparison		Anxious	Not anxious	Within group comparison		Anxious	Not anxious	Within group comparison		Anxious	Not Anxious	Within group comparison	
	*n*; (%)	*n*; (%)	Total	*P*-value	*n*; (%)	*n*; (%)	Total	*P* – value	*n*; (%)	*n*; (%)	Total	*p*-value	*n*; (%)	*n*; (%)	Total	*p*-value
T_0_	13; (50.0)	13; (50.0)	26	0.7	13; (44.8)	16; (55.2)	29	0.59	6; (28.6)	15; (71.4)	21	0.51	6; (20.7)	23; (79.3)	29	1
T_1_	10; (45.5)	12; (54.5)	22		11; (37.9)	18; (62.1)	29		2; (18.2)	9; (81.8)	11		6; (20.7)	23; (79.3)	29	
COVID-19 specific phobia	Presence	Absence	Within group comparison		Presence	Absence	Within group comparison		Presence	Absence	Within group comparison		Presence	Absence	Within group comparison	
	*n*; (%)	*n*; (%)	Total	*P*-value	*n*; (%)	*n*; (%)	Total	*P* – value	*n*; (%)	*n*; (%)	Total	*p*-value	*n*; (%)	*n*; (%)	Total	*p*-value
T_0_	10; (38.5)	16; (61.5)	26	0.42	16; (55.2)	13; (44.8)	29	0.79	9;(42.9)	12; (57.1)	21	0.38	8; (27.6)	21; (73.4)	29	0.008*
T_1_	11; (50.0)	11; (50.0)	22		15; (51.7)	14; (48.3)	29		3; (27.3)	8; (72.7)	11		11; (37.9)	18; (62.1)	29	

*n*: frequency; %: percentage; T_0 _= baseline session; T_1 _= follow-up session; *p* ≤ 0.05*.

## Discussion

This study provides a valuable understanding of the acceptability of telemental health interventions among pregnant women in Qatar. Pregnant women in our sample showed low acceptance of telemental health interventions: 50% of the eligible pregnant women did not want to participate. Our study reported a 35% attrition rate, whereas one-to-one intervention usually is associated with lower attrition rates, around 25%. The video component and mental health stigma may be the perceived barriers that led to a higher rejection rate. Our findings contradict pregnant women’s views in Western countries, where they show high acceptance of receiving mental health intervention as it allows them to achieve a positive pregnancy experience (Evans et al., 2020; Waters et al., 2020).

When examining time zero (baseline level), the sociodemographic, pregnancy-related characteristics and psychosocial outcomes did not show any statistical differences between the two arms (intervention group vs. control group), indicating no differences in risk factors at baseline. Exploring baseline characteristics is essential as we can adjust for any significant risk factor between intervention and control groups if found, but it was similar.

When examining the efficacy of the telemental low-intensity psychosocial intervention, we revealed a significant effect between the intervention and control groups in decreasing antenatal anxiety symptoms among pregnant women. Our findings are consistent with a previous study that revealed that internet-based cognitive behavioural therapy (ICBT) produces moderate to significant effect size reductions in anxiety (Hedges’ *g* = 0.76) and no significant differences in reducing depression (Hedges’ *g* ≤ 0.35) among pregnant women (Loughnan et al., 2019). However, this previous study lacked an active control.

We did not find a statistically significant difference in antenatal depression amongst pregnant women in the treatment vs. control arm. Our finding is similar to a study that showed a difference between pregnant women receiving ICBT and the standard treatment when the outcome was assessed through EPDS. Even though the EPDS screening tool is reliable and sensitive, it might not be a suitable instrument to assess antenatal depression in interventional studies; when EPDS failed to detect differences, another tool, Montgomery Åsberg Depression Rating Scale Self-report version (MADRS-S), was able to detect differences (Forsell et al., 2017). However, a meta-analysis conducted among the perinatal population revealed a moderate effect for depression (Hedges’ *g* = 0.60; 95% CI 0.43–0.78) and anxiety (Hedges’ *g* = 0.54; 95% CI 0.24–0.85). Notably, this meta-analysis must be interpreted carefully as most of the included studies were conducted during the postnatal period (Loughnan et al., 2019).

The concept of low-intensity CBT is a revolution in mental healthcare and expanded recently in response to the growing demands of mental health treatment. Low-intensity CBT overlaps designed to be the first level of intervention, utilizing self-help materials and less frequent follow-up sessions compared to the standard CBT. An apparent inconsistency in the utilization of the terms low-intensity CBT and brief traditional high-intensity CBT; such variation in the labelling of interventions limit proper comparison and aggregation of data (Shafran et al., 2021).

In the pre-COVID-19 time, some studies applied the concept of low-intensity CBT. To specify, a short-term group-based CBT revealed a significant decline in antenatal depressive scores in the intervention arm compared with receiving usual care after three weeks of follow-up (Tandon et al., 2014). Furthermore, a personalized four weeks’ mindfulness-based intervention showed a significant decline in depressive scores among pregnant women (Sun et al., 2022). Moreover, a preliminary meta-analysis of interventional studies (*n* = 13) that had short follow-up periods and less intensive sessions revealed that CBT's pooled mean effect size on perinatal anxiety was small (*k* = 7; *d* = 0.49; 95% CI: 0.08–0.91). Despite favouring the CBT arm (Q1 = 30.13, *p* < 0.001), it is crucial to highlight that the small between-group effect sizes (*d* = 0.49) possibly indicate that CBT may not be more effective than non-active control conditions (Maguire et al., 2018).

The published studies differ significantly from our study regarding the research question, design, inclusion criteria, and control availability. For example, a prospective longitudinal cohort study of an eight-week treatment period investigated intervention feasibility among pregnant women with severe fear of childbirth and who had not previously experienced birth. Thus, different inclusion criteria were used (Nieminen et al., 2016). Furthermore, another study investigated the ICBT treatment effect; however, the published RCT included pregnant women with a severe major depressive disorder who were on psychotropic medications (Kim et al., 2014). In contrast, we excluded patients receiving any psychotropic medications as it could be a confounder to the proposed intervention.

COVID-19-specific phobia levels have been assessed in other cross-sectional studies, and they were found to be associated with increased antenatal anxiety and depression (Fitzpatrick et al., 2020; Giesbrecht et al., 2022). However, we did not find an interventional survey targeting this outcome. Furthermore, our data shows that low-intensity psychosocial support does not have a beneficial effect in reducing COVID-19-specific phobia among pregnant women in Qatar.

We also conducted a within-group analysis; we found a significant difference in antenatal depression scores within the control group. The placebo effect could be the possible justification, which is a response to any therapy regardless of the physiological effect. However, the intervention's actual effect is the percentage effect in the intervention group minus the percentage effect due to the placebo. Additionally, within the intervention group, there was a significant improvement in COVID-19-specific phobia, but only through intention-to-treat analysis, not protocol analysis (Lewis et al., 2017).

## Strengths and limitations

To the best of our knowledge, this is the first study to examine the impact of maternal low-intensity psychosocial telemental interventions on antenatal depression, anxiety, and COVID-19-specific phobia during the COVID-19 pandemic in Qatar. One of the strengths of the study is that a psychologist guided our intervention, which may result in a better impact on health outcomes than a non-guided one. Usually, teleconsultation limits the ability to see patient body language and hampers provider assessment of patient self-care. Therefore, we chose a synchronous video telemental health application (Baumeister et al., 2014).

We applied randomization and double blinding to eliminate selection bias in assigning intervention. Furthermore, we used concealment to reduce performance and ascertainment bias after randomization (Campbell et al., 2000), and the data collectors were trained in phase one of the study to prevent measurement bias. Additionally, reliable screening tools were used to assess outcomes. Finally, the longitudinal design preserves temporality and the causation effect. We computed Hedges’ g value of the continuous measurements and NNT for the dichotomous variables to ensure adequate effect size estimation with precise confidence interval measurement (McGough & Faraone, 2009). However, this study does not come without limitations. The sample size is small, limiting the generalization of the results, and the dropout rate of the participants was higher than desired. Thus, these limitations may compromise the external validity of the results. Furthermore, there is a probability of having misclassification bias as we utilized screening tools to identify psychosocial outcomes, giving four possible choices [true and false positives, true and false negatives].

## Conclusions

This trial is the first telemental low-intensity psychosocial intervention RCT performed among pregnant women in Qatar. The study shows that telemental low-intensity psychosocial intervention could effectively reduce antenatal anxiety symptoms. However, additional RCTs are needed to replicate the evidence.

## Data Availability

The datasets used and/or analysed during the current study are available from the corresponding author on reasonable request. CC BY 4.0 license.
